# Recall and Reconsolidation of Contextual Fear Memory: Differential Control by ERK and Zif268 Expression Dosage

**DOI:** 10.1371/journal.pone.0072006

**Published:** 2013-08-16

**Authors:** Antoine Besnard, Jocelyne Caboche, Serge Laroche

**Affiliations:** 1 Institut National de la Santé et de la Recherche Médicale UMRS 952, Physiopathologie des Maladies du Système Nerveux Central, Paris, France; 2 Centre national de la recherche scientifique UMR7224, Physiopathologie des Maladies du Système Nerveux Central, Paris, France; 3 UPMC, Université Paris 6, Paris, France; 4 Université Paris-Sud, Centre de Neurosciences Paris-Sud, Orsay, France; 5 Centre national de la recherche scientifique UMR 8195, Orsay, France; Nathan Kline Institute and New York University Langone Medical Center, United States of America

## Abstract

Compelling evidence points to the existence of independent cellular processes involved in the consolidation and reconsolidation of memory. For instance, a double dissociation has been reported between hippocampal Extracellular-Regulated Kinase-1/2 (ERK1/2) activity being necessary for contextual fear conditioning (CFC) consolidation but not reconsolidation. Conversely, hippocampal expression of the immediate early gene *Zif268* is necessary for CFC reconsolidation but not consolidation. Since we previously reported that ERK1/2 controls the transcription of *Zif268* in the hippocampus, we examined the precise role of ERK1/2 activity and *Zif268* gene expression dosage in CFC memory processing. For this, we first assessed performance of *Zif268* homozygous and heterozygous mutant mice in a CFC paradigm. Whereas *Zif268*−/− mice displayed a deficit of both consolidation and reconsolidation, *Zif268*+/− mice displayed a selective deficit of reconsolidation only, therefore pointing to the relationship between *Zif268* gene expression dosage and CFC memory processing. *Zif268* gene expression dosage interfered with the reconsolidation process if and only if CFC memory was relatively recently encoded and directly reactivated. Furthermore, CFC memory strengthening previously reported to involve Zif268 expression in the hippocampus was spared in *Zif268*+/− mice. Finally, blocking ERK1/2 activity prior to CFC retrieval prevented the deficit of reconsolidation observed in *Zif268*+/− mice. Collectively, these results highlight a tight relationship between *Zif268* gene expression dosage and CFC memory processing. They also suggest that ERK1/2 activity upon CFC memory recall is necessary for its retrieval, a prerequisite for its reactivation and subsequent reconsolidation.

## Introduction

Contextual fear conditioning (CFC) is a well-established paradigm to study the neural mechanisms of emotional learning and memory. The task consists of a brief training episode that pairs a physical context with a shock-US [1], resulting in a long-lasting memory of the context-US association, the formation of which engages the hippocampus and amygdala [1]–[3]. The stabilization of the memory trace following learning is a time-dependent process referred to as consolidation [4]. Once established, memories are thought to persist in an inactive state and to return back to an active state upon recall [5]. During recall, reactivation of the memory trace can however destabilize the original memory in order to allow the incorporation of additional features into the original memory trace [6]. A reconsolidation process then follows to restabilize the updated version of the memory into an inactive memory available for further recall [5]. Since the rehabilitation of the reconsolidation theory in fear memory paradigms [7], a great deal of effort has been placed on determining whether or not reconsolidation is a simple repetition of consolidation [5]. Although certain similarities between the two processes have been highlighted, there also is evidence to support the existence of mutually exclusive mechanisms controlling the consolidation and reconsolidation processes [6], [8]–[10]. For example, hippocampal Extracellular-Regulated Kinase-1/2 (ERK1/2) activity is necessary for CFC acquisition and consolidation [11], but not reconsolidation [12]–[14]. Conversely, partial hippocampal knockdown of the immediate early gene, *Zif268* affects CFC reconsolidation but not initial storage [15]. Interestingly, ERK1/2 activity [12], [16] as well as *Zif268* transcription [17] and protein expression [15], [16] were shown to be increased in the hippocampus following CFC retrieval, a situation where memory reactivation initiate reconsolidation of CFC memory. Since ERK1/2 activity can control activity-dependent transcription of *Zif268* [18], these observations raised the question of the precise role of ERK1/2 and Zif268 in CFC memory processing. To investigate this issue, we trained wild-type, homozygous (*Zif268*−/−) and heterozygous (*Zif268*+/−) mutant mice in a trial unique CFC paradigm and examined post-learning and post-recall performance to investigate the relationship between *Zif268* gene expression dosage and CFC memory processing. Whereas *Zif268*−/− mice displayed a deficit of CFC consolidation, *Zif268*+/− mice showed a selective deficit of reconsolidation in this task. *Zif268* gene expression dosage interfered with reconsolidation if and only if CFC memory was relatively recently encoded and directly reactivated. Furthermore, CFC memory strengthening previously reported to involve Zif268 expression in the hippocampus was spared in *Zif268*+/− mice. Finally, blocking ERK1/2 activity prior to CFC retrieval prevented the deficit of reconsolidation observed in *Zif268*+/− mice. Collectively, these results highlight a tight relationship between *Zif268* gene expression dosage and CFC memory processing. We propose that upon CFC memory recall, ERK1/2 activation is an early molecular event required for CFC memory retrieval, followed by *Zif268* regulation required for memory restabilization.

## Materials and Methods

### Mice

A total of 144 mice were used in this study. *Zif268* knockout mice were generated as described previously [19] and backcrossed onto a C57BL/6J background for 24 generations. Age-matched (2–8 month old) *Zif268*+/+, *Zif268*+/− and *Zif268*−/− male littermate mice were used for behavioral experiments. Mice were maintained in a 12 h light/dark cycle in stable conditions of temperature (22°C) and humidity (60%), in groups of 4 to 5 with food and water *ad libitum*. Testing was performed during the light phase of the cycle. Three days before the experiments, mice were briefly handled each day. Mice were sacrificed by CO2 inhalation at the end of the experiments. All efforts were made to decrease the number of animals used in each experiment and to minimize suffering. Experimental protocols were approved by the ethics committee of the French Agriculture and Forestry Ministry for handling animals (decree 87/849, license B75-05-22).

### Drugs

The MEK inhibitor SL327 (Sigma-Aldrich, St. Quentin Fallavier, France) was dissolved in 100% DMSO and administrated by intraperitoneal injection (30 mg/kg; 2 ml/kg) 1 hour prior to the experiment. Control mice received the same volume of DMSO without SL327 (vehicle).

### Contextual Fear Conditioning (CFC)

#### General procedure

Mice were trained in conditioning chambers (17.5×17.5×15 cm) that had stainless steel rod floor through which footshocks could be delivered. Training consisted of placing mice in the chamber and delivering an unsignaled footshock (2 sec duration; 0.7 mA) 150 sec later. Mice were returned to their home cages 30 sec after the footshock. Memory was assessed as the percentage of time mice spent freezing when replaced in the training context. Freezing behavior (defined as complete lack of movement, except for respiration) was assessed at 5 sec intervals over a 300 sec period [20]. For the memory strengthening experiment, freezing behavior was measured before each shock-pairings over a 150 sec period.

#### Experiment 1

**Figure 1:**
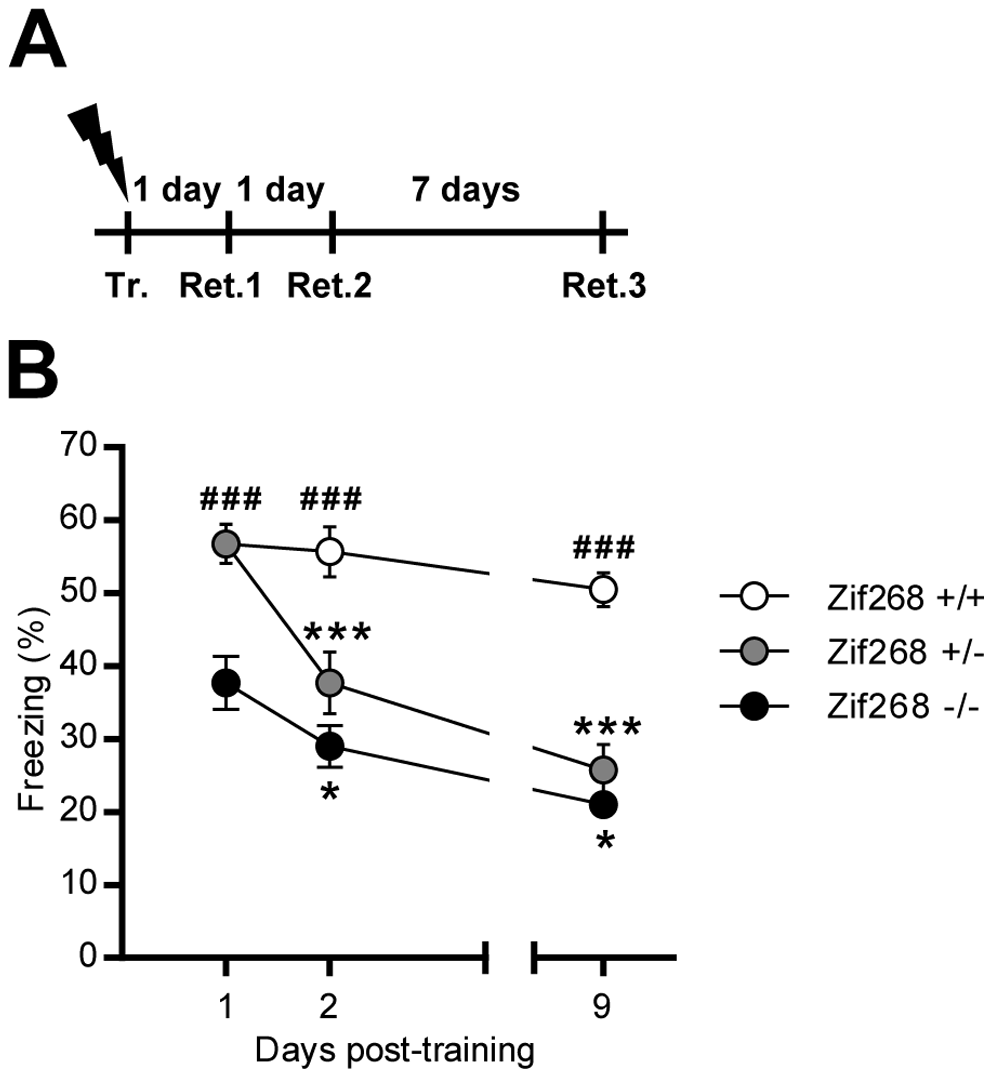
Effect of Zif268 gene dosage on CFC consolidation and reconsolidation. **A)** Experimental design. *Zif268*+/+, *Zif268*+/− and *Zif268*−/− mice were trained (Tr.) and retrieval sessions were conducted 1 day (Ret.1), 2 days (Ret.2) and 9 days (Ret.3) after training. **B)** Freezing behavior was measured in *Zif268*+/+ (white circles), *Zif268*+/− (grey circles) and Zif268−/− (black circles) mice during each retrieval session. *Zif268*−/− mice show a deficit of consolidation whereas *Zif268*+/− mice display a selective impairment of reconsolidation. Data are means ± SEM; n = 10 mice per group. ^###^p<0.001, *Zif268*+/− or −/− versus Zif268+/+; *p<0.05; ***p<0.001 present versus past retrieval.

Effect of *Zif268* gene dosage on CFC consolidation and reconsolidation (Fig. 1). *Zif268* wild-type (+/+), heterozygous (+/−) and homozygous mutant (−/−) mice were trained and 5 min re-exposure sessions to the training context were conducted 1 day (Retrieval 1), 2 days (Retrieval 2) and 9 days (Retrieval 3) after training.

#### Experiment 2

**Figure 2:**
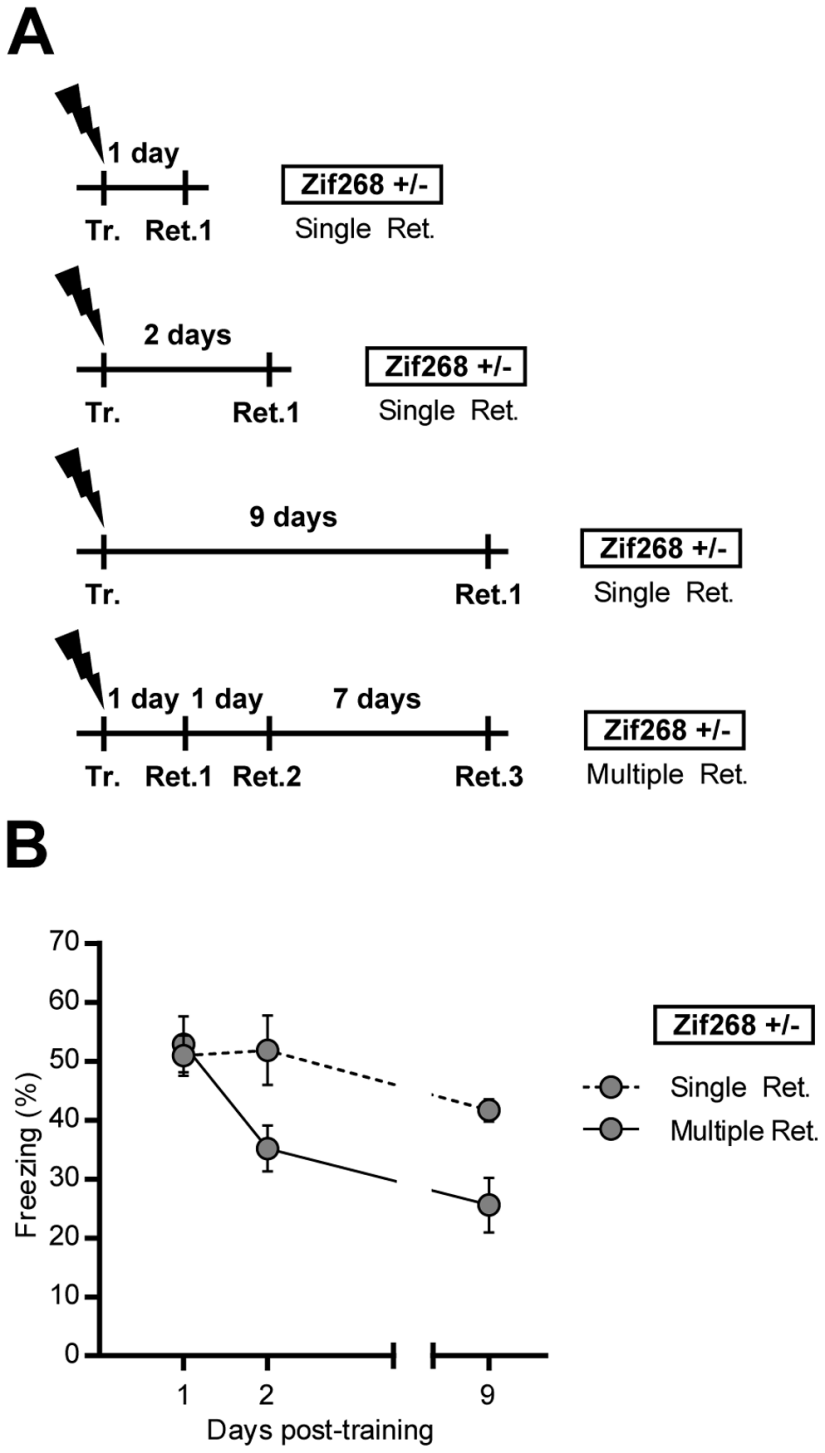
Effect of Zif268 gene dosage on reactivated and non-reactivated CFC memories. **A)** Experimental design. *Zif268*+/− mice were trained (Tr.) and retrieval sessions were conducted 1 day (Ret.1), 2 days (Ret.2) and 9 days (Ret.3) after training. **B)** Freezing behavior was measured in *Zif268*+/− mice during each test session. One group was repeatedly submitted to the 3 retrieval sessions (Multiple retrieval – grey circles, solid line) and three groups were submitted only once to one of the 3 retrieval sessions (Single retrieval – grey circles, dotted line). The deficit of reconsolidation observed in *Zif268*+/− mice is dependent on re-exposure to the context. Data are means ± SEM; n = 8–10 mice per group.

Effect of *Zif268* knockdown on reactivated and non-reactivated CFC memories (Fig. 2). Four groups of *Zif268*+/− mice were trained and 5 min re-exposure session to the training context were conducted 1 day (Retrieval 1), 2 days (Retrieval 2) and 9 days (Retrieval 3) after training. Mice of one group were submitted to the three retrieval sessions, whereas mice in three independent groups were submitted to only one of the retrieval sessions.

#### Experiment 3

**Figure 3:**
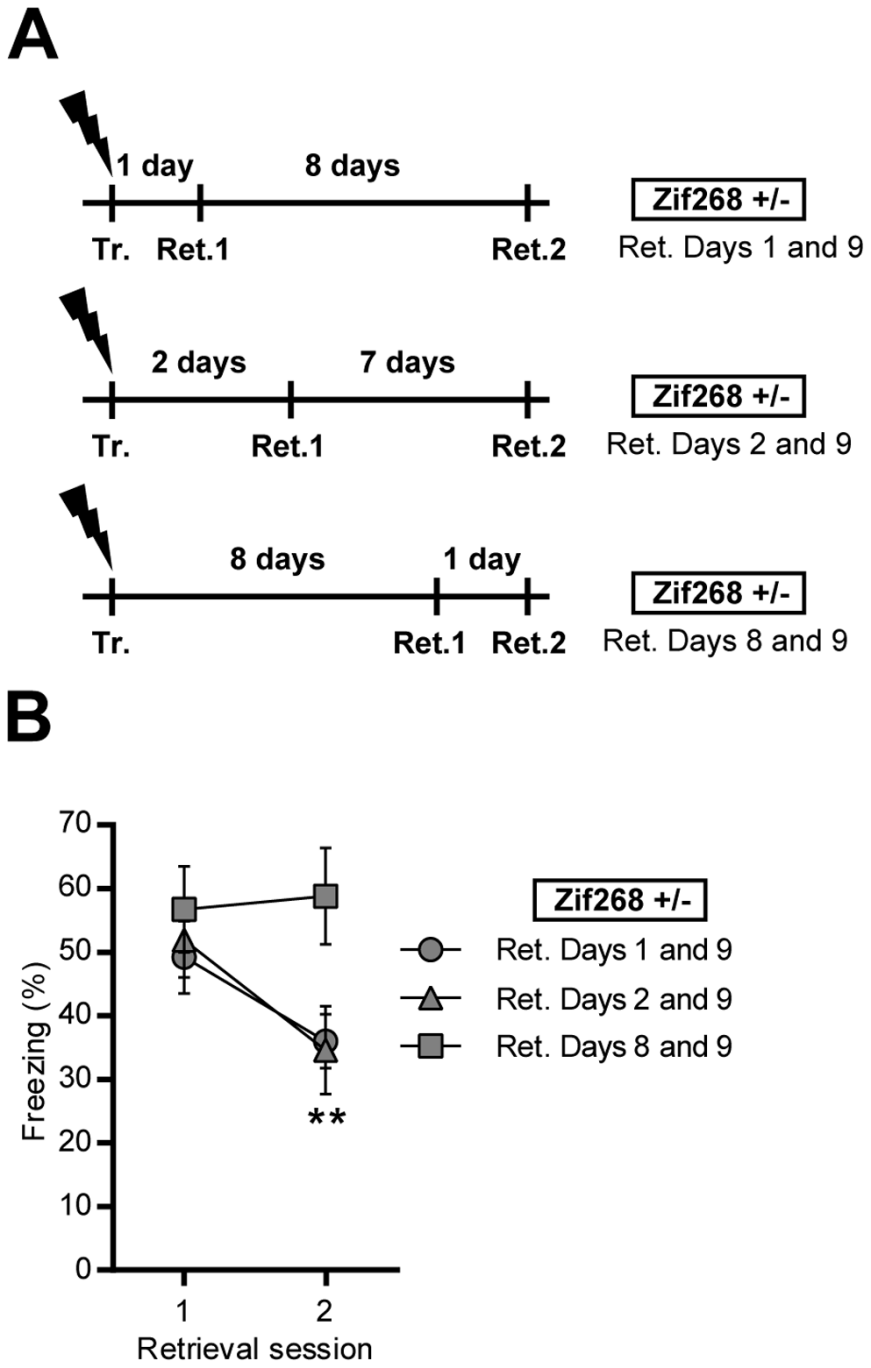
Effect of Zif268 gene dosage on the reconsolidation of 1 and 8 days-old CFC memories. **A)** Experimental design. *Zif268*+/− mice were trained (Tr.) and retrieval sessions (Ret.1 and Ret.2) were conducted in independent groups 1 and 9 days, 2 and 9 days or 8 and 9 days after training. **B)** Freezing behavior was measured in *Zif268*+/− mice during each test session 1 and 9 days (grey circles), 2 and 9 days (grey triangles) or 8 and 9 days (grey squares) following training. The reconsolidation deficit observed in *Zif268*+/− mice is not observed when the memory has been encoded 8 days earlier. Data are means ± SEM; n = 9–10 mice per group. **p<0.01, present versus past retrieval.

Effect of *Zif268* knockdown on the reconsolidation of 8 day-old CFC memory (Fig. 3): Three independent groups of *Zif268*+/− mice were trained and 5 min re-exposure sessions (Retrieval 1 and 2) to the training context were conducted 1 and 9 days, 2 and 9 days or 8 and 9 days after training.

#### Experiment 4

**Figure 4:**
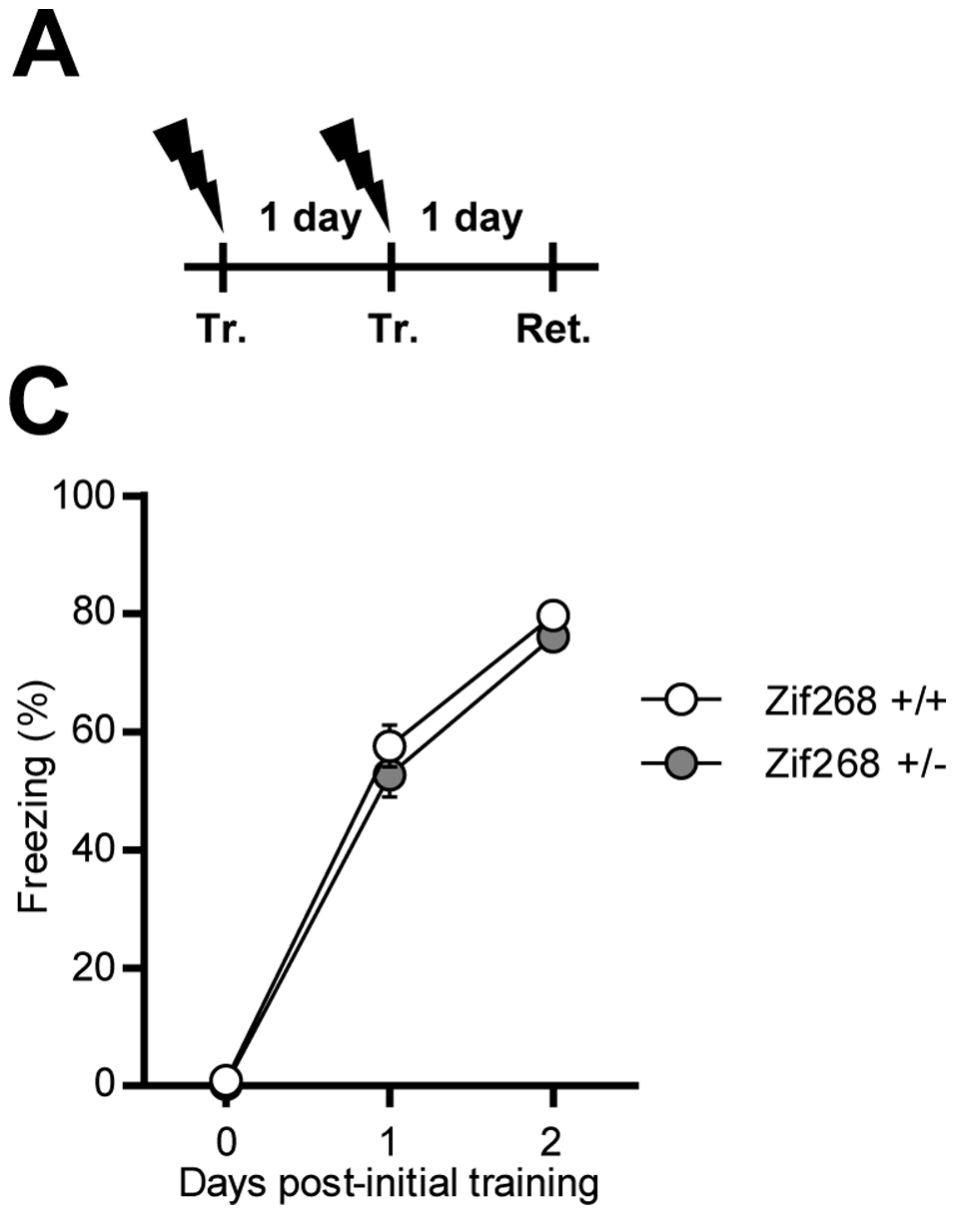
Effect of Zif268 gene dosage on CFC memory strengthening. **A)** Experimental design. *Zif268*+/+ and *Zif268*+/− mice were trained for two successive days (Tr.1 and Tr.2) and a retrieval (Ret.) session was conducted on subsequent day. **B)** Freezing behavior was measured in *Zif268*+/+ (white circles) and *Zif268*+/− (grey circles) mice before each shock-pairings as well as during the retrieval session. *Zif268* knockdown did not affect the strengthening of CFC memory. Data are means ± SEM; n = 11 mice per group.

Effect of *Zif268* knockdown on CFC memory strengthening (Fig. 4). *Zif268*+/+ and *Zif268*+/− mice were trained and a second training session was conducted 1 day later. The mice were submitted to a 5 min re-exposure session to the training context (Retrieval) on the subsequent day. Freezing behavior was measured before each shock-pairing (150 sec) and during the retrieval session.

#### Experiment 5

**Figure 5:**
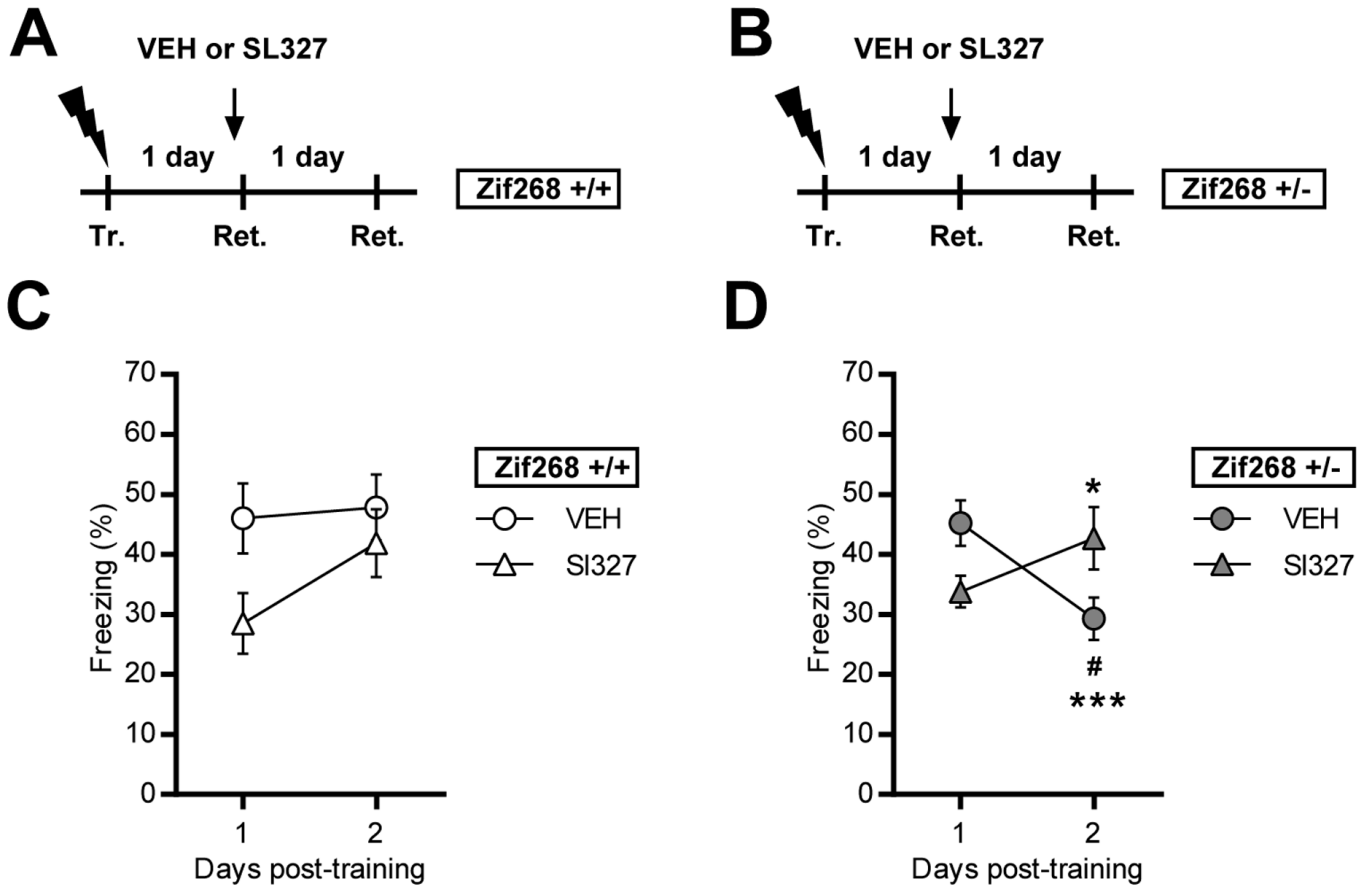
Effect of MEK inhibition on Zif268 gene dosage-dependent impairment of CFC reconsolidation. **A–B)** Experimental designs. *Zif268*+/+ mice and *Zif268*+/− mice were trained (Tr.) and on subsequent day, vehicle (white diamonds) or SL327 (black diamonds) were administrated 1h before retrieval (Ret.). An additional retrieval session was conducted on the next day. **C–D)** Freezing behavior was measured in vehicle (VEH, circles) and MEK inhibitor (SL327, triangles) treated *Zif268*+/+ (white) and *Zif268*+/− (grey) mice during each retrieval session. SL327 transiently impaired freezing behavior of *Zif268*+/+ when injected before the first retrieval session. SL327 had a protecting effect against the deficit of reconsolidation observed in *Zif268*+/− mice. Data are means ± SEM; n = 9–10 mice per group in C; n = 8–9 mice per group in D. ^#^p<0.05, SL327 versus Vehicle; *p<0.05; ***p<0.001, present versus past retrieval.

Effect of MEK inhibition on *Zif268* knockdown-dependent impairment of CFC reconsolidation (Fig. 5): *Zif268*+/+ and *Zi268*+/− mice were trained, and the vehicle or SL327 were administrated 1 hour before a 5 min re-exposure session to the context conducted 1 day (Retrieval 1) after training. An additional retrieval session was conducted 2 days (Retrieval 2) after training.

### Statistics

Data are presented as the mean ± SEM. Data (Experiment 1,4,5) were analyzed using mixed factor two-way ANOVA (repeated measure over time). Data from Experiment 2 were analyzed using mixed factor two-way ANOVA (non-repeated measure over time). Data from Experiment 3 were analyzed using paired Two-tailed Student’s t-test. Two-way ANOVA were followed by *post- hoc* comparisons using Bonferroni test only when the interaction between factors was statistically significant [21]. In all cases, significance threshold was set at p<0.05.

## Results

### Effect of Zif268 Gene Dosage on CFC Consolidation and Reconsolidation

Compelling evidence supports the notion that Zif268 expression is critical for the reconsolidation of CFC [15] as well as for that of auditory cued-fear conditioning [22], [23]. In light of the complex relationship between *Zif268* gene expression dosage and behavioral performance observed across distinct type of memories [24]–[26], we took advantage of *Zif268* mutant mice to assess the relationship between *Zif268* expression and CFC memory processing. We trained *Zif268*+/+, *Zif268*+/− and *Zif268*−/− mice and challenged freezing performance 1, 2 and 9 days following training (Fig. 1A). Clear differences in performance were observed between the three groups across the three retention tests (mixed factor two-way ANOVA (repeated measure over time): effect of time, F_(2,54) = _72.38, p<0.001; effect of genotype, F_(2,27) = _21.76, p<0.001; interaction, F_(4,54) = _12.21, p<0.001; followed by post-hoc comparisons (Bonferroni test); Fig. 1B). On the first retrieval session 24 h after training, freezing performance of *Zif268*+/+ and *Zif268*+/− mice was undistinguishable. *Zif268*−/− mice, however, showed a clear deficit in freezing behavior 24 h after training (Fig. 1B). This consolidation deficit in CFC in *Zif268*−/− mice, which display a complete loss of Zif268 expression in the whole brain are consistent with previous reports in as much as Zif268 expression in the amygdala was previously reported to be required for the consolidation of a contextual fear conditioning memory [27], albeit not the hippocampus [15]. A subsequent test conducted 1 day later, however, revealed an important decrease in freezing behavior in *Zif268*+/− mice when compared to their performance the previous day and to the performance of *Zif28*+/+ mice (p<0.001 in each case; Fig. 1B). Interestingly, freezing performance of *Zif268*−/− mice also decreased after the first retrieval session. No spontaneous recovery was observed when mice were retested 7 days later. There was even a further decrease in performance of *Zif268*+/− and *Zif268*−/− mice when compared to the previous retrieval session, whereas there was minimum, if any, decrease of performance in *Zif268*+/+ mice, suggesting that the memory loss at the preceding session in *Zif268*+/− and *Zif268*−/− mice was not complete and that each additional retrieval session further increased the deficit. These results indicate that *Zif268* gene expression dosage is an important factor controlling CFC memory. They show that total absence of *Zif268* in the whole brain results in a deficit in CFC consolidation, whereas half the complement of Zif268 does not affect the initial storage of CFC memory but impairs its reconsolidation following recall.

### Effect of Zif268 Knockdown on Reactivated and Non-reactivated CFC Memories

Reactivation of the original memory has previously been reported to constrain memory reconsolidation [28]. We therefore investigated the influence of memory reactivation on memory reconsolidation in *Zif268*+/− mice. We trained different groups of *Zif268*+/− mice and challenged freezing performance 1, 2 and 9 days following training (Fig. 2A). One group was tested at the three post-training intervals and three groups were tested once at each interval. Overall analysis of performance by two-way ANOVA (non-repeated measure over time) revealed a significant effect of time (F_(2,45) = _8.99, p<0.001), of procedure (F_(1,45) = _8.46, p<0.01) with no significant interaction (F_(2,45) = _3.07, NS) (Fig. 2B). In the group of *Zif268*+/− mice submitted to multiple retrieval sessions, freezing behavior decreased between the first and second retrieval sessions, and no spontaneous recovery was observed on day 9, thus replicating the above results. In contrast, when *Zif268*+/− mice were submitted to retrieval only once, freezing performance was maintained at a high level over the three training-to-retrieval intervals, with only a slight non-significant decay between day 2 and 9. These results indicate that the deficit of reconsolidation observed in *Zif268*+/− mice after memory recall is dependent on the reactivation of the original memory and therefore does not reflect spontaneous extinction of the original memory.

### Effect of Zif268 Knockdown on the Reconsolidation of Recent and 8 Days-old CFC Memory

The influence of the age of the memory has previously been defined as a boundary condition for memory reconsolidation [10]. We thus investigated the influence of memory age on freezing performance in *Zif268*+/− mice (Fig. 3A). Data were analyzed using paired Two-tailed Student’s t-test (Fig. 3B). We trained three groups of *Zif268*+/− mice, a first group was submitted to retrieval sessions on days 1 and 9, a second group on days 2 and 9 and a third group on days 8 and 9 post-conditioning. We observed a strong decrease in freezing performance on the second retrieval session on day 9 only in the groups of *Zif268*+/− mice previously submitted to a retrieval test on days 1 or 2 (p<0.01 in each case), but not when the first retrieval session was given on day 8 post-conditioning (Fig. 3B). These results confirm that *Zif268* gene expression dosage is critical for the reconsolidation of a recently encoded CFC memory. They also indicate that reconsolidation of an older (8 days) memory is less sensitive to partial down-regulation of Zif268 expression.

### Effect of Zif268 Knockdown on CFC Memory Strengthening

Since hippocampal Zif268 expression is also involved in the strengthening of CFC memory [29], we assessed performance of *Zif268*+/+ and *Zif268*+/− mice in this paradigm involving two training sessions, 1 day apart (Fig. 4A). Analysis of performance using mixed factor two-way ANOVA (repeated measure over time) revealed a significant effect of time (F_(2,40) = _636.2, p<0.001) but no significant effect of genotype (F_(1,20) = _1.93, NS) or interaction (F_(2,40) = _0.48, NS) (Fig. 4B). Both on the second reinforced training session and on the retention test 1 day after the last training session, *Zif268*+/+ and *Zif268*+/− mice displayed similar freezing performance. These results suggest that half the complement of Zif268 is not sufficient to prevent the strengthening of CFC memory.

### Effect of MEK Inhibition on Zif268 Knockdown-dependent Impairment of CFC Memory Reconsolidation

ERK1/2 is rapidly activated in several brain structures, including hippocampal subregions and amygdala nuclei, upon CFC recall [16] and in the amygdala following cued-fear memory recall [30]. As the ERK1/2 signaling cascade can control activity-dependent expression of Zif268, we investigated the involvement of ERK1/2 in CFC retrieval and subsequent reconsolidation in relation to *Zif268* gene expression dosage. For this, *Zif268*+/+ and *Zif268*+/− mice were injected with vehicle or SL327, an inhibitor of the ERK1/2 upstream kinase MEK, 1 h before recall of a previously established contextual fear memory (Fig. 5A–B). In *Zif268*+/+ mice, we found that MEK inhibition 1 h prior to recall impaired performance at the recall test, compared to vehicle-injected mice (Fig. 5C). Interestingly, freezing performance recovered to the level of vehicle-injected mice when SL327-injected *Zif268*+/+ mice were re-tested on the subsequent day (mixed factor two-way ANOVA (repeated measure over time): effect of time, F_(1,17) = _7.38, p<0.05; effect of treatment, F_(1,17) = _2.55, NS; interaction, F_(1,17) = _4.22, NS; Fig. 5C). In *Zif268*+/− mice, injection of the vehicle had no specific effect on performance: freezing was high on the first retrieval test, and low on the subsequent retrieval test 1 day later (Ret 1 vs Ret2: p<0.05; Fig. 5D), replicating the above impairment of post-retrieval long-term memory. In contrast, MEK inhibition impaired *Zif268*+/− freezing performance on the first retrieval session 1 h after SL237 injection, as it did in *Zif268*+/+ mice; however this impairment was transient as performance of the mice recovered to a high level of freezing on the second test session. Data were analyzed using mixed factor two-way ANOVA (repeated measure over time: effect of time, F_(1,15) = _2.79, NS; effect of treatment, F_(1,15) = _0.03, NS; interaction, F_(1,15) = _35.54, p<0.001; post-hoc comparisons (Bonferroni test): Ret1 *vs.* Ret2, p<0.001; SL327 *vs.* vehicle, p<0.05; Fig. 5D). Altogether, these results confirm previous reports pointing to the involvement of ERK1/2 in CFC retrieval [12] and suggest a role for ERK1/2 activity in the reactivation of CFC memory upon recall as seen in both *Zif268*+/+ and *Zif268*+/− mice. Furthermore, the absence of reconsolidation deficit in *Zif268*+/− mice treated with the MEK inhibitor before recall indicates that MEK inhibition before memory retrieval, by preventing reactivation of the original memory, renders this memory trace immune to reconsolidation.

## Discussion

In the present work, we combined genetic and pharmacological strategies to dissect out the role of ERK1/2 and Zif268 in contextual fear memory processing. Over the past decade, expression of Zif268 mRNA was shown to be rapidly induced in the hippocampus and amygdala following contextual and cued fear conditioning [31], [32]. Similar observations were made for Zif268 protein expression levels [16], [23], [33], which confirmed that Zif268 is rapidly and transiently expressed following fear learning. In a recent report, an increase in Zif268 binding to its DNA ERE consensus sequence in the hippocampus was observed following inhibitory avoidance learning, thus suggesting a functional role for this transcriptional regulator [34]. In parallel, several approaches established the critical role of Zif268 in both long-term synaptic plasticity [24] and the stabilization of long-term memories [35]–[37]. Targeted deletion of the *Zif268* gene was shown to prevent the consolidation of spatial memory, conditioned taste aversion, social transmission of food preference and object or object-place recognition memory [24], [25]. Conversely, using a gain-of-function strategy in an inducible transgenic mouse, *Zif268* overexpression in the forebrain was reported to strengthen conditioned taste aversion memory [38]. In fear memory, however, the functional role of Zif268 in consolidation appears less clear. Antisense oligodeoxynucleotides injection, which partially knockdowns Zif268 expression levels, failed to affect retention of contextual fear memory when injected in the hippocampus [15], but impaired both long-term contextual [27] and cued [23] fear memories when infused into the amygdala. Our results using a full knockout of *Zif268* in the whole brain cannot address the issue of structure-specificity. However, they clearly confirm the importance of Zif268 brain expression for long, but not short-term stabilization/consolidation of CFC memory.

Concomitant to the description of Zif268 induction following fear learning, several studies reported that Zif268 mRNA expression can also be increased following fear memory retrieval in structures such as the hippocampus and amygdala [17], nucleus accumbens and prefrontal cortex [39]. In line with these observations, fear memory recall was also shown to be associated with an increase in Zif268 protein expression in the hippocampus [15], [16], [34] and amygdala [40]. Importantly, alternative strategies for inhibiting Zif268 supported the notion that Zif268 is required for memory reconsolidation. Zif268 loss-of-function was shown to impair reconsolidation of object and object-place recognition memory [41], [42] and Zif268 knockdown in the hippocampus was shown to impair CFC reconsolidation [15]. In light of the complex relation between *zif268* gene expression dosage and behavioral performance across distinct type of memories [24]–, we took advantage of *Zif268* homozygous and heterozygous mutant mice to assess the relationship between *Zif268* gene expression and CFC memory. While *Zif268* homozygous knockout mice displayed a striking impairment of both memory consolidation and reconsolidation, *Zif268* heterozygous mice displayed a selective impairment of reconsolidation. These data indicate that *Zif268* gene expression dosage is an important feature of the different phases of CFC memory processing and highlight the higher vulnerability of reconsolidation to Zif268 expression levels, compared to post-training consolidation. Interestingly, we recently reported that CFC retrieval triggers in the dentate gyrus a lower magnitude of Zif268 expression than CFC training [16]. This could be in part linked to the nature of the stimuli presented during CFC training (footshock associated with the context) and CFC retrieval (re-exposure to the context in the absence of the footshock). However, in light of the higher sensitivity of reconsolidation to Zif268 partial down-regulation, it is tempting to speculate a relationship between Zif268 expression dosage and fear memory processing. These findings echo previous reports suggesting that reconsolidation is more sensitive than consolidation to interfering treatments such as hypothermia [43] or pharmacological manipulations [44]–[47].

Since the original demonstration that protein synthesis is re-engaged following recall to restabilize memory traces and make them available for further recall [7], further analysis of the phenomenon provided firm evidence that the reconsolidation process only occurs when the memory is directly reactivated [28]. We therefore investigated the influence of memory reactivation induced by contextual re-exposure to the environment explicitly associated with the footshock. One group was repeatedly submitted to retrieval and three groups were submitted to retrieval only once. An impairment of performance was observed in *Zif268* heterozygous mice undergoing multiple, but not single retrieval sessions. These results indicate that the deficit of reconsolidation induced by *Zif268* gene expression dosage is dependent on the reactivation of the original memory and therefore does not reflect memory decay over time.

The influence of the age of the memory on subsequent reconsolidation is an important matter of debate. If reconsolidation of recently encoded memories was consistently reported, some studies indicated that remote memories remain susceptible to reconsolidation [48]–[51], while others suggested that older memories become less amenable to reconsolidation [52]–[54]. Our results showing impaired post-retrieval long-term memory in *Zif268* heterozygous mice 1 or 2 days, but not 8 days post-training suggest that *Zif268* gene expression dosage is only critical for the reconsolidation of recently encoded CFC memory. A previous study using intra-hippocampal injection of the protein synthesis inhibitor anisomycin reported that the hippocampus remains involved in the reconsolidation of 7 to 45 days-old CFC memories [48]. The absence of reconsolidation of an 8-day old memory reported here thus does not imply that CFC memory becomes rapidly immune to reconsolidation, but likely reflects the fact that our approach resulted in only a 50% reduction in the expression of a single gene, as opposed to the more global inhibition of protein synthesis.

In an elegant report, CFC memory strengthening by repeated training was shown to involve a reconsolidation process depending on hippocampal Zif268 expression [29]. We therefore assessed performance of *Zif268* heterozygous mice in this paradigm. We found no effect of *Zif268* gene expression dosage on CFC memory strengthening. This result suggests that a 50% loss of Zif268 is not sufficient to prevent the strengthening of CFC memory. Comparatively, the missense oligodeoxynucleotide strategy used in the abovementioned study [29] resulted in a 66% decrease of Zif268 expression as measured in hippocampal area CA1 [15]. Altogether, these results unveil the tight relationship between *Zif268* gene expression dosage and the processing of CFC memory, in which memory consolidation, reconsolidation and strengthening display distinct degrees of sensitivity to Zif268 downregulation.

Activation of ERK1/2 is at the crossroads of fear related memory consolidation [55] and retrieval [12]. Importantly, ERK1/2 activation in the amygdala is required for the consolidation and reconsolidation of cued fear memory [30], [56]. In contrast, ERK1/2 activity in the hippocampus was consistently reported to be dispensable for CFC reconsolidation following a short (2–3 min) contextual reminder [12]–[14]. However, we recently found that ERK1/2 is activated in the hippocampus upon CFC recall [16] and we previously demonstrated that ERK1/2 is involved in the induction of *Zif268* expression [18]. Thus, the apparent lack of ERK1/2 involvement in the reconsolidation of CFC prompted us to gain further insights into this phenomenon by exploring the specific contribution of ERK1/2 in CFC memory retrieval and reconsolidation in relation to *Zif268* gene expression dosage. We first observed that inhibition of the ERK1/2 upstream kinase MEK prior to recall decreases recall performance in both wild type and *Zif268* heterozygous mice, confirming the early description by Chen and colleagues (2005) that ERK1/2 activity is critical for the retrieval of a CFC memory.

Upon a second retention test, however, we observed a recovery of freezing performance in both wild type and *Zif268* heterozygous mice. These results imply, first, that transient inhibition of ERK1/2 activity does not permanently alter the ability to recall a CFC memory and second, that ERK1/2 activity is not involved in the reconsolidation of CFC memory, as previously reported [12]–[14]. However, whereas *Zif268* heterozygous mice injected with the vehicle were impaired on the second retention test, replicating the above reconsolidation deficit, Zif268 heterozygous mice injected with the MEK inhibitor, despite their impairment on the first retention test, showed normal performance on the second retention test 1 day later. Thus, MEK inhibition during recall protected the mice against the deleterious effect of *Zif268* gene knockdown on memory reconsolidation. These results suggest that ERK1/2 inhibition during the retention test, by preventing retrieval and reactivation of the memory, avoided destabilization of the memory and consequently suppressed the need for its reconsolidation, therefore alleviating the need for Zif268 activation. Mechanistically, the contribution of ERK1/2 activity to retrieval implies a rapid action of the kinase. Besides the known transcriptional role of ERK1/2 activation [57], ERK1/2 can control multiple molecular partners in different cellular compartments leading to rapid post-translational modifications affecting neuronal excitability and synaptic transmission, independently of transcriptional programs [58]. For example, ERK can modulate dopamine synthesis [59] and the Kv4.2 primary pore-forming subunit of the potassium channel [60] that plays a critical role in shaping the electrical response of neurons [61]–[63]. ERK1/2 can also activate Synapsin I and decrease synapsin-actin bundling, increasing the likelihood of vesicle fusion and therefore of transmitter release [64]–[66], and contribute to AMPA receptors trafficking during synaptic plasticity [67], [68]. Selective intervention on these mechanisms could be a promising approach in deciphering the signaling mechanisms involved in mediating the effect or ERK1/2 activation on memory retrieval.

In the past years, a great deal of effort has been placed on determining whether or not reconsolidation is a repetition of consolidation. This considerable effort unveiled unexpected anatomical, cellular and molecular signatures specific to memory reconsolidation. Here, we present the view that *Zif268* gene expression dosage can also distinguish both processes. We also propose that common molecular events, such as ERK1/2 activation, might recruit different molecular partners involved in the retrieval and subsequent restabilization of a fear memory. To gain further insights into the role of ERK1/2 signaling in selective memory processes, one strategy for future research would be to interfere with selective ERK molecular partners, while preserving its global activity.
